# Clinical Indicators Suggesting Dapsone-Induced Methemoglobinemia in Dermatology Outpatients: A Pilot Retrospective Cohort Study

**DOI:** 10.7759/cureus.111188

**Published:** 2026-06-20

**Authors:** Jerene Mathews, Anju Thomas, Pooja Lekshmi S, Haritha B Meda, Febin Ashraf

**Affiliations:** 1 Dermatology, All India Institute of Medical Sciences Bhopal, Bhopal, IND; 2 Dermatology, Believers Church Medical College Hospital, Kuttapuzha, IND

**Keywords:** adverse drug reaction, arterial blood gas, dapsone, methemoglobinemia, methemoglobin (methb), pulse oximetry, retrospective cohort study

## Abstract

Background

Dapsone is used widely in dermatology as an anti-inflammatory agent for diseases including lichen planus, dermatitis herpetiformis, bullous pemphigoid, and sub-corneal pustular dermatosis (SCPD). Anemia and methemoglobinemia are known hematologic adverse effects of dapsone. These occur through distinct mechanisms and therefore develop independently of each other. Mild-to-moderate methemoglobinemia produces non-specific symptoms such as fatigue, exertional dyspnea, headache, and postural syncope that may affect daily functioning, impair treatment compliance, and lead to unnecessary evaluation when dapsone is not recognized as the cause. Biochemical confirmation requires arterial blood gas (ABG) measurement with co-oximetry, not routinely performed in dermatology outpatient settings.

Objectives

In this pilot retrospective cohort study, we aimed to estimate the prevalence and describe the clinical pattern of findings consistent with dapsone-induced methemoglobinemia in a dermatology outpatient clinic, evaluate the feasibility of a simple symptom- and pulse-oximetry-based screening strategy, and generate hypotheses for a prospective study with systematic methemoglobin (MetHb) measurement.

Methods

This study was conducted at a tertiary care dermatology outpatient clinic in central Kerala, India, over an 18-month period. Of a total of 41 patients prescribed dapsone during the study period, 28 had at least one follow-up visit while on dapsone and were included in the study. Clinical findings consistent with methemoglobinemia were defined as new-onset symptoms (fatigue, exertional dyspnea, headache, or postural syncope) or peripheral oxygen saturation (SpO2) at or below 94% on pulse oximetry, temporally correlated with dapsone initiation and not attributable to anemia or other comorbid illness. ABG MetHb levels were available in four patients only. Non-parametric tests were used for all comparisons due to the small sample size. All analyses were considered exploratory and hypothesis-generating given the pilot design.

Results

Of 28 patients followed up, 12 (43%) had clinical findings consistent with dapsone-induced methemoglobinemia, nine did not have any of the findings, and seven had insufficient data to be assessed against the clinical definition. Elevated MetHb levels were confirmed on ABG in four patients, all of whom had been classified as consistent with methemoglobinemia on clinical grounds, providing preliminary support for the feasibility of symptom and pulse-oximetry-based screening. No evaluated risk factor reached statistical significance. No patient required treatment beyond dapsone discontinuation.

Conclusion

This pilot study identifies a substantial burden of clinical findings consistent with dapsone-induced methemoglobinemia in a dermatology outpatient setting, comparable to rates in immunocompromised cohorts. Mild-to-moderate symptoms, easily misattributed to other causes, were the dominant presentation and carry real implications for symptom burden and treatment compliance. The four ABG-confirmed cases suggest, but do not confirm, the validity of clinical screening. Prospective studies are needed to evaluate the diagnostic performance and clinical utility of systematic screening strategies in dermatology outpatients receiving dapsone.

## Introduction

Dapsone (4,4'-diaminodiphenylsulfone) is a sulfone antibiotic used widely in dermatology for inflammatory diseases including lichen planus, dermatitis herpetiformis, bullous pemphigoid, sub-corneal pustular dermatosis (SCPD), urticarial vasculitis, and hidradenitis suppurativa [1]. Its primary toxic metabolite, dapsone hydroxylamine, causes two distinct hematologic adverse effects, hemolysis and methemoglobinemia, through separate pathways [1-3]. Glucose-6-phosphate dehydrogenase (G6PD) activity protects red cells against hemolysis, whereas cytochrome b5 reductase (CYB5R) protects against methemoglobinemia by reducing methemoglobin (MetHb) back to functional hemoglobin. These defences are independent: partial CYB5R deficiency or M-globin variants predispose to methemoglobinemia even when G6PD is normal, so G6PD testing cannot serve as a surrogate for methemoglobinemia screening [1,2,4,5]. Cytochrome P450 (CYP450) enzymes generate the metabolite; CYP450 inducers increase its formation, and inhibitors such as cimetidine reduce it [6], and toxicity is further influenced by dapsone dose, concurrent oxidizing drugs, and antioxidant intake such as vitamins C and E [1,7,8].

Severe methemoglobinemia presents with breathlessness, cyanosis, and SpO₂ that is disproportionately low and unresponsive to supplemental oxygen. Mild-to-moderate methemoglobinemia, however, produces non-specific symptoms such as fatigue, headache, exertional dyspnea, and postural syncope that may not be apparent at rest and are readily attributed to other causes [1]; cyanosis may be absent, particularly in dark-skinned individuals [9]. These symptoms may affect daily functioning, impair adherence to dapsone therapy, and, when dapsone is not recognized as the cause, lead to misattribution and unnecessary evaluation; for example, a fall in oxygen saturation (SpO₂) prompting pulmonary workup, or fatigue prompting cardiac or endocrine investigation, while the drug effect goes unrecognized. The severity of symptoms depends not solely on MetHb levels but also on total hemoglobin, rate of onset, and coexisting cardiorespiratory disease [10]. As a result, MetHb levels do not reliably predict clinical burden or need for intervention. Biochemical confirmation requires ABG measurement with co-oximetry, which is invasive, expensive, and not routinely performed in dermatology outpatient settings.

Published incidence data are limited to immunocompromised populations. Subramaniam et al. (2010) reported symptomatic methemoglobinemia in 22.2% of patients with hematologic malignancy in Australia [11]. In a pediatric renal transplant cohort in the United States, Hindka et al. (2021) found elevated MetHb in 50% of patients, with symptomatic disease in 65% of those affected; fatigue was the dominant symptom in 90% [10]. Among adult renal transplant recipients in the United Kingdom, Mitsides et al. (2014) reported an incidence of 46%, with symptomatic disease in 50% of those affected [12]. Esbenshade et al. (2011) reported elevated MetHb in 19.8% of patients with hematologic malignancy, with a median MetHb of 9.0% (IQR 3.5-22.4%) and a dose-dependent effect observed [13]. O'Neal studied 22 patients on dapsone for *Pneumocystis jiroveci* prophylaxis in HIV, and found elevated MetHb in 31.8% and a median MetHb of 1.3% (IQR 0.6-2.5%) [14]. To the best of our knowledge, no comparable data exist for a dermatology outpatient setting in India or globally, and no established screening protocol exists for this setting.

In this pilot, hypothesis-generating study, we aimed to estimate the prevalence and describe the clinical pattern of findings consistent with dapsone-induced methemoglobinemia in a dermatology outpatient clinic, evaluate the feasibility of a simple symptom- and pulse-oximetry-based screening strategy, and generate hypotheses for a prospective study with systematic biochemical MetHb measurement.

Parts of this study were previously presented as a poster at the European Academy of Dermatology and Venereology (EADV) Annual Congress 2024, Amsterdam, The Netherlands, and as a free paper at the Dermatology and Allied Specialities Summit (DAAS) 2024, New Delhi, India.

## Materials and methods

Study design and setting

This was a pilot retrospective cohort study conducted at the dermatology outpatient clinic of Believers Church Medical College Hospital, a tertiary care center in Thiruvalla, Kerala, India, over an 18-month period. This study was approved by the Institutional Ethics Sub Committee, Believers Church Medical College Hospital (IEC Study No. IEC/2023/11/374, dated September 7, 2023). As this was a medical records-based retrospective study, the requirement for individual written informed consent was waived by the ethics committee.

Study population

A total of 41 patients were prescribed dapsone by the dermatology outpatient department during the study period. Of these, 13 patients had no follow-up visit while on dapsone; six did not attend for reasons unrelated to dapsone (primarily distance of residence from the hospital), six for unknown reasons, and one due to intolerance to dapsone with no further details available. The remaining 28 patients, who had at least one follow-up visit while on dapsone, formed the study cohort.

Clinical case definition

Given the retrospective design and the routine non-availability of ABG measurement in this outpatient setting, a clinical case definition was used as the primary outcome. Clinical findings were classified as consistent with dapsone-induced methemoglobinemia if the patient had one or more of the following symptoms or signs, new in onset after dapsone initiation and not explained by anemia or a comorbid condition (such as cardiac failure or bronchial asthma). Symptoms included new-onset fatigue, exertional dyspnea, headache, or postural syncope. Signs included tachycardia, tachypnea, cyanosis, or a fall in SpO2 to 94% or below on pulse oximetry.

These symptoms were selected because they are the recognised clinical manifestations of mild-to-moderate methemoglobinemia described in standard references [1], corresponding to the symptom profile expected in an ambulatory setting. Features of severe disease (marked cyanosis, altered sensorium, seizures) were not included as these are rare and unlikely to be missed. All patients with any of the symptoms or signs recorded in electronic medical records were assessed for the time of onset with respect to dapsone intake. SpO₂ was measured by handheld pulse oximetry on room air during routine outpatient visits. Supplemental oxygen was not routinely administered to patients with low SpO2 unless they were symptomatic. 

All patients on dapsone had measurements of hemoglobin levels at baseline and on follow-up as part of the clinic protocols. So, anemia as a cause of symptoms was easily assessed based on the serial hemoglobin levels. All patients had their comorbidities (especially cardio-respiratory) also documented at the initiation of dapsone. Most of the patients who developed symptoms/signs had undergone evaluation for potential causes by appropriate specialists like a pulmonologist, cardiologist, or hematologist for the same. In one patient, dyspnea was due to bronchial asthma and was relieved with bronchodilators. In seven patients, available data were insufficient to apply or exclude the case definition: five patients did not have a documented SpO2, and in two patients, the symptoms had not been adequately evaluated. In four patients, ABG measurement with co-oximetry had been performed as part of routine clinical care; a MetHb level above 2% was considered elevated. These four cases were used to assess concordance between the clinical case definition and biochemical confirmation.

Data collection

Data were extracted retrospectively from medical records. Variables collected included age, sex, body weight, indication for dapsone, maximum dapsone dose (mg/kg/day), comorbid illnesses, G6PD levels, baseline and lowest recorded Hb (Hb in g/dl), baseline and lowest recorded SpO2, new-onset symptoms on dapsone, examination findings, concurrent medications with oxidizing potential, antioxidant supplementation (vitamins C and E), ABG MetHb levels where available, and reason for dapsone discontinuation. (See Appendices for a full list of drugs associated with methemoglobinemia.

Statistical analysis

Due to the small sample size, non-parametric tests were used for all statistical comparisons. Continuous variables are reported as median and IQR and were compared using the Mann-Whitney U test. Categorical variables were analyzed using Fisher's exact test. A p-value less than 0.05 was considered statistically significant. Missing data were not imputed. The seven patients with insufficient data to apply the case definition were retained in the prevalence denominator but excluded by listwise deletion from the comparative risk-factor analysis (12 versus nine patients). All analyses are considered exploratory and hypothesis-generating given the pilot design. All statistical analyses were performed using R version 4.6.0 (R Foundation for Statistical Computing, Vienna, Austria, https://www.R-project.org/).

## Results

Patient characteristics

**Figure 1:**
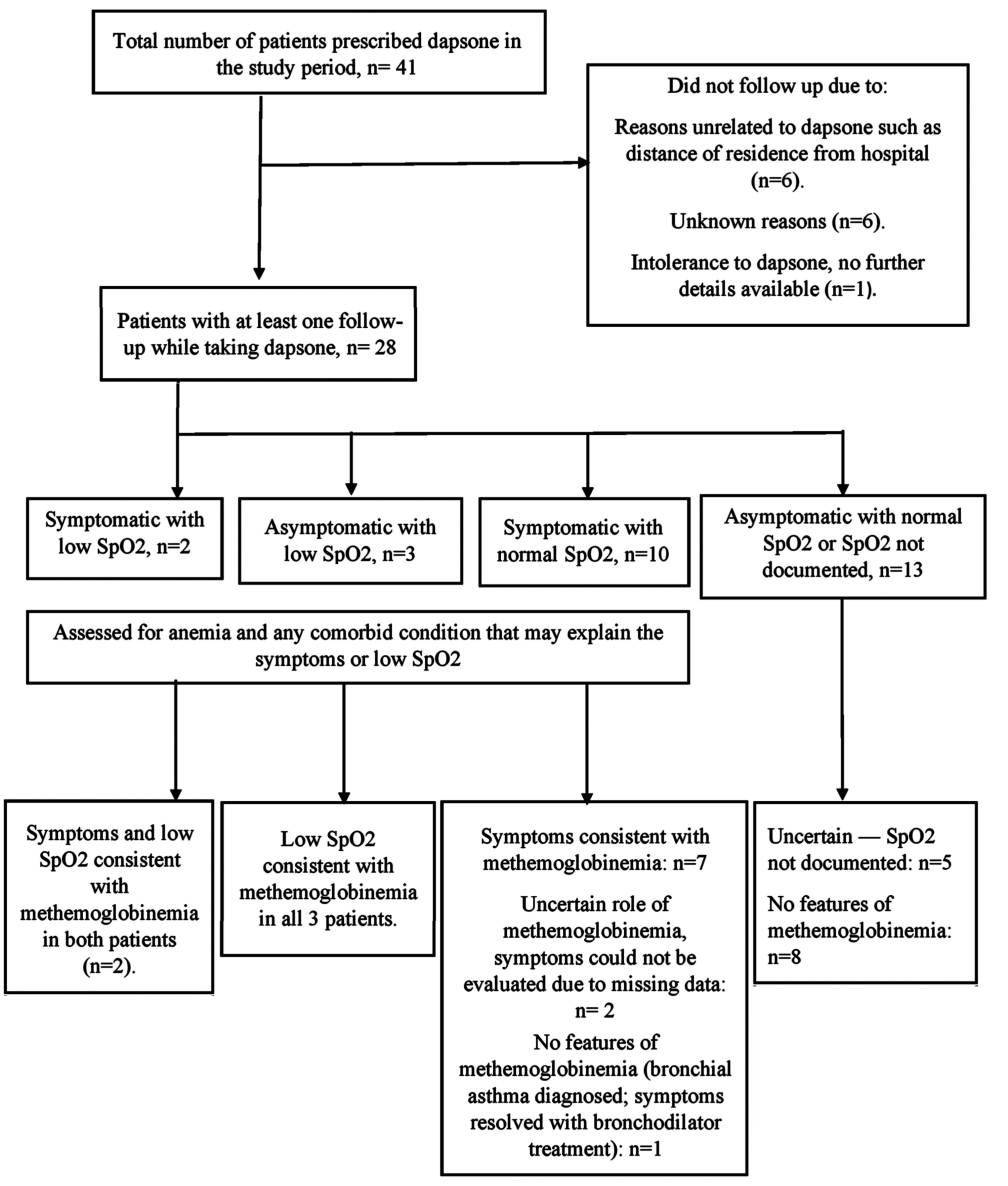
Flowchart showing the selection and evaluation of the study cohort

A total of 28 patients with at least one follow-up visit on dapsone were included in the study (Figure 1). Patients were stratified by presenting features and assessed for anemia and comorbid conditions that may account for symptoms or low SpO2. Clinical findings consistent with methemoglobinemia were identified in 12 patients (43%); nine patients (32%) did not meet the case definition; and seven patients (25%) could not be evaluated due to insufficient data. Of the 28 patients, five had low SpO2 at or below 94% detected on pulse oximetry. The evaluation process of the study participants is summarised in Figure 1.

The median age of the participants was 55.5 years (range: 2-87 years), and 19 (68%) were female. The median dapsone dose was 1.52 mg/kg/day (range: 0.42-2.2 mg/kg/day). The indications for dapsone are summarized in Table 1; lichen planus was the most common indication (12 patients, 42.8%).

**Table 1:** Indication for dapsone in study cohort (N=28)

No.	Indication	Frequency (Percentage)
1	Lichen planus	12 (42.8)
2	Dermatitis herpetiformis and dermatitis herpetiformis-like dermatitis	4 (14.2)
3	Sub-corneal pustular dermatosis (SCPD)	3 (10.7)
4	Bullous pemphigoid	2 (7.1)
5	Urticarial vasculitis	2 (7.1)
6	Others: recurrent erythema multiforme, hidradenitis suppurativa, recurrent idiopathic aphthosis, drug-induced lichenoid eruption, pemphigus foliaceous (one patient each)	5 (17.9)

Baseline characteristics of the 13 patients without follow-up were also analysed and were found to be broadly similar to the included cohort (median age 27 years, range 6-47; 10/13 female; median baseline haemoglobin 13.1 g/dl). Indications were comparable, with lichen planus most common. One patient discontinued dapsone because of intolerance; the remainder did not attend follow-up, six for reasons unrelated to dapsone and six for unknown reasons.

Biochemical confirmation in four patients

ABG with co-oximetry was available in four patients, all of whom met the clinical case definition prior to ABG review. MetHb levels were 3.3%, 4.2%, 6.2%, and 7%, respectively, confirming elevated MetHb in all four (Table 2). Three of these four had low SpO2 on pulse oximetry (89%, 92%, and 94%, respectively); one had SpO2 of 98% but had fatigue and exertional dyspnea. This concordance between clinical findings and biochemical confirmation suggests, but does not confirm, that symptom and pulse-oximetry-based screening identifies true cases of methemoglobinemia. But as ABG was done only in four patients (based on clinical judgement), the performance of the screening method could not be adequately assessed. 

**Table 2:** Characteristics of the four patients with biochemically confirmed methemoglobinemia on arterial blood gas analysis. All values in this table are taken directly from patient records; MetHb >2% on arterial blood gas co-oximetry was considered elevated. MetHb: methemoglobin; SpO2: peripheral oxygen saturation; F: female

Patient	Age (years)/sex	Indication	Dose (mg/kg/day)	MetHb (%)	SpO2 (%)	Symptoms	Outcome
1	70/F	Lichen planus	1.26	3.3	94	Low SpO2; exertional dyspnea present at baseline	Dapsone continued; both anemia and methemoglobinemia contributed to presentation
2	56/F	Sub-corneal pustular dermatosis	0.90	4.2	98	Fatigue, exertional dyspnea	Dapsone continued; anemia also contributed to presentation
3	64/F	Urticarial vasculitis	1.80	6.2	89	None — incidental SpO2 finding	Dapsone stopped at patient request following counseling about low SpO2
4	51/F	Dermatitis herpetiformis	1.30	7.0	92	Fatigue, low SpO2	Dapsone stopped due to symptoms affecting activities of daily living.

**Figure 2:**
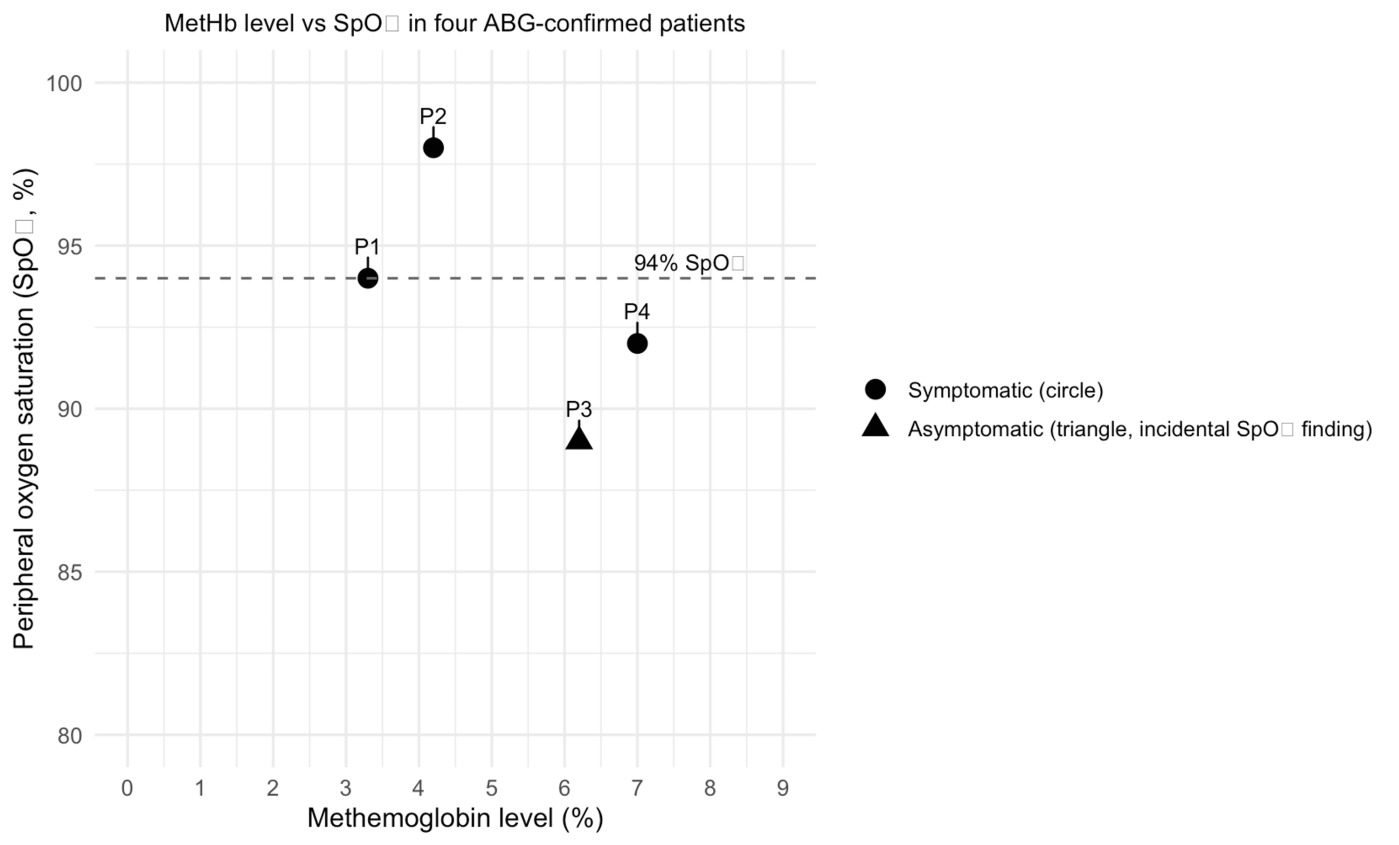
Relationship between methemoglobin (MetHb) level and peripheral oxygen saturation (SpO2) in the four patients with arterial blood gas confirmation of methemoglobinemia. Each data point represents one patient; filled circles indicate symptomatic patients and open triangles indicate asymptomatic patients. The dashed horizontal line indicates SpO2 of 94%, the threshold used in the clinical case definition. n=4 The figure is illustrative only and is not statistically powered. The image was generated using R version 4.6.0 (R Foundation for Statistical Computing, Vienna, Austria, https://www.R-project.org/)

The patient with the highest MetHb (7.0%) was symptomatic, while the patient with MetHb at 6.2% was entirely asymptomatic, illustrating the poor correlation between MetHb level and symptom severity (Figure 2).

Prevalence of clinical findings consistent with methemoglobinemia

Of the 28 patients, 12 (43%; 95% CI 26.5-60.9%) had clinical findings consistent with dapsone-induced methemoglobinemia. Nine patients (32%) did not have any such clinical findings. Seven patients (25%) were classified as uncertain due to insufficient data about symptoms or SpO2 (Figure 1). The most common symptom was fatigue, present in seven patients (25%). Exertional dyspnea was present in four (14%), headache in two (7%), and postural syncope in one (4%). Five patients reported more than one symptom. No patient had clinical signs of tachycardia, tachypnea, or cyanosis on examination at rest, signs typically associated with moderate-to-severe methemoglobinemia.

**Figure 3:**
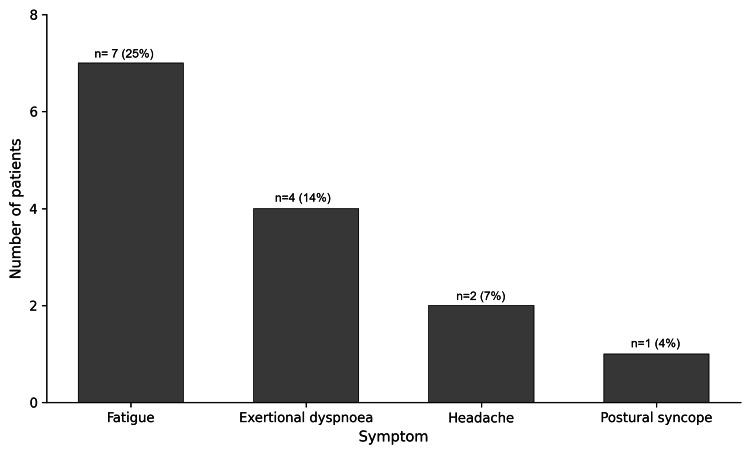
Frequency of new-onset symptoms among the study cohort (N=28) Generated using R version 4.6.0 (R Foundation for Statistical Computing, Vienna, Austria, https://www.R-project.org/)

The full follow-up cohort of 28 patients was retained as the denominator for the calculation of the prevalence of methemoglobinemia. The seven patients with insufficient data were not excluded, as excluding them, five of whom were asymptomatic without documented SpO₂, would preferentially remove likely non-cases and overestimate the burden of methemoglobinemia (12/21 = 51%). The group of 12 (with clinical findings of methemoglobinemia) was compared with the nine patients (without the same clinical findings) for analysis of risk factors of methemoglobinemia. Of the 12 patients, two had both new-onset symptoms and low SpO2, three had low SpO2 without symptoms, and seven had new-onset symptoms alone (Figure 1). Low SpO₂ (≤94%) was detected in 5/28 (17.9%, 95% CI 7.9-35.6%).

Clinical impact: symptom burden and treatment decisions

Mild-to-moderate symptoms were the dominant presentation. Seven patients had fatigue, and four had exertional dyspnea: symptoms that affected daily functioning and were identified as new and burdensome in the clinical record. In at least three patients, symptoms were initially attributed to other causes (anemia, bronchial asthma, or post-COVID small airway disease) before dapsone was considered, illustrating the risk of misattribution when clinicians are not alerted to methemoglobinemia as a differential. One patient with an isolated low SpO2 and no symptoms elected to stop dapsone after counseling, suggesting that even asymptomatic findings, if not explained, can drive unnecessary treatment decisions. One patient demonstrated a dose-dependent effect, tolerating dapsone at 50 mg per day without symptoms but developing findings consistent with methemoglobinemia at 100 mg per day, suggesting dose optimization as a potential management strategy.

Management and outcomes

Dapsone was discontinued in four of the 28 patients, and no patient required methylene blue therapy or any treatment other than discontinuation. In the patient with MetHb 7.0% (P4 in Figure 2), fatigue and low SpO₂ (92%) resolved after stopping dapsone, with symptom resolution and normal SpO₂ documented at the next outpatient review approximately one month later. In the asymptomatic patient with MetHb 6.2% and low SpO₂ (89%) (P3 in Figure 2), follow-up SpO₂ and MetHb levels and the timeline to resolution were not documented. A third patient (ABG not performed) with new-onset fatigue, exertional dyspnoea, and persistently low SpO₂ (94%) had a cardiac cause excluded by normal echocardiography; cessation was driven mainly by symptoms, and post-cessation SpO₂ and the timeline to resolution were not documented. In a fourth patient with new-onset fatigue and exertional dyspnoea but normal SpO₂ (ABG not performed), symptoms were reported to improve after stopping dapsone, though the timeline to resolution was not documented.

Risk factor analysis

Risk factors were compared between 12 patients with clinical findings consistent with methemoglobinemia and nine patients without (Table 3).

**Table 3:** Risk factors in patients with clinical findings consistent with methemoglobinemia versus those without NOTE: Seven patients with insufficient data for case definition classification were excluded. IQR: interquartile range; G6PD: glucose-6-phosphate dehydrogenase; Hb: hemoglobin. *p<0.05 was considered statistically significant. Non-parametric tests were used throughout due to the small sample size. †Mann-Whitney U test. ‡Fischer's exact test.

Variable	Clinical findings consistent with methaemoglobinaemia (n=12)	Clinical findings not consistent with methaemoglobinaemia (n=9)	Test statistic	p-value*
Age in years, median (IQR)	53 (18)	50 (29)	U = 57.5	0.83†
Sex	Male 2, Female 10	Male 4, Female 5	Fisher’s exact test	0.11‡
Comorbid illness	Anaemia: 1; bronchial asthma: 2; post-COVID small airway disease: 1; hypothyroidism: 3; diabetes mellitus: 3; hypertension: 3; dyslipidaemia: 2; coronary artery disease: 1	Anaemia: 2; bronchial asthma: 0; other chronic lung disease: 0; hypothyroidism: 1; diabetes mellitus: 2; hypertension: 2; dyslipidaemia: 2; coronary artery disease: 1	-	
G6PD levels	Normal in 9; not available in 3	Normal in 7; not available in 2	-	
Dose of dapsone in mg/kg/day, median (IQR)	1.52 (0.33)	1.40 (0.35)	U = 58.5	0.78†
Intake of other drugs with oxidising effects	None	Aspirin in one patient; isosorbide dinitrate in another	Fisher’s exact test	0.17‡
Intake of vitamins C and E with dapsone	Both vitamins C and E in 9; vitamin C only in 3	Both vitamins C and E in 8; vitamin C only in 1	Fisher’s exact test	0.60‡
Fall in Hb on dapsone in g/dl, median (IQR)	1.75 (1.60)	1.80 (0.47)	U = 43.0	0.73†

Age, sex, G6PD status, dapsone dose, concurrent oxidizing drugs, antioxidant supplementation, and hemoglobin fall did not differ significantly between the two groups. Notably, nine of the 12 patients in the consistent group had normal G6PD levels, confirming that normal G6PD status does not protect against this complication. The statistical non-significance of all evaluated risk factors is likely due to the small sample size. Clinically relevant associations may be detected in a larger prospective study.

## Discussion

This pilot study found that 43% of patients (12/28; 95% CI 26.5-60.9%) in a dermatology outpatient clinic developed clinical findings consistent with dapsone-induced methemoglobinemia. Prevalence was calculated using the full cohort of 28 patients rather than only the 21 with complete data, since the study aimed to characterize the overall burden of clinical findings across the cohort. Restricting the denominator to the 21 fully evaluated patients would have inflated the estimate to 51% and further reduced an already small sample. Using 28 as the denominator keeps the estimate conservative.

Thirteen patients prescribed dapsone did not follow up after initiation of dapsone and could not be assessed. Their baseline characteristics were broadly similar to those of the included cohort. However, the reasons for non-attendance were unknown in six, and one discontinued because of intolerance. Patients who develop adverse effects of a therapy may be more likely to change healthcare providers or default from follow-up, a recognised source of differential loss to follow-up (attrition bias) [15]. As this would preferentially remove symptomatic patients from our cohort, it would tend to bias the observed prevalence downward, reinforcing that our 43% estimate is conservative.

Because the study was retrospective, MetHb levels from ABG were available only in four patients, all of whom had elevated MetHb, and all of whom had already been identified as clinically consistent cases before the ABG results were seen. Because ABG was obtained only in clinically suspected patients, this concordance is subject to selection bias and does not represent a formal measure of diagnostic accuracy. It offers preliminary, hypothesis-generating support for a symptom- and SpO₂-based screening approach pending confirmation in a prospective study.

Although the small sample yields a wide confidence interval (95% CI 26.5-60.9%), the point estimate of 43% is consistent with rates reported in immunocompromised cohorts: 46% in adult renal transplant recipients in the United Kingdom [12] and 50% in pediatric renal transplant patients in the United States [10] both fall within this interval, while the 22.2% symptomatic rate (four of 18 tested) reported in patients with hematologic malignancy in Australia sits just below the lower bound [11]. The 19.8% reported by Esbenshade et al., who applied a stricter biochemical threshold [13], lies similarly below the interval. The overlap of our wide interval with these rates is consistent with a pharmacological class effect rather than a complication limited to immunocompromised patients, though the small sample precludes a firm conclusion.

What stands out most from this cohort is that the clinical picture was dominated by mild-to-moderate disease. No patient had tachycardia, tachypnea, or cyanosis, signs typically seen with moderate-to-severe methemoglobinemia. Patients were often normal at rest; it was on exertion or with daily activity that fatigue and dyspnea became apparent. This matters clinically because these are precisely the symptoms most likely to go unnoticed or be attributed to something else. In our cohort, at least three patients had their symptoms initially blamed on anemia, bronchial asthma, or post-COVID small airway disease. This pattern, where the drug effect is real but invisible within the usual clinical framework, is what makes systematic screening so important. When dapsone is not on the differential, the patient ends up with unnecessary investigations or treatments while the actual cause goes uncorrected.

The absence of classical signs also has a direct implication for how we monitor these patients: relying on clinical examination alone will miss most cases of mild methemoglobinemia. Pulse oximetry adds useful information; it detected low SpO₂ at or below 94% in five patients (5/28; 95%CI 7.9-35.6%), including one who was entirely asymptomatic. Esbenshade et al. (2011) found SpO2 below 95% in 78% of patients with elevated MetHb [13], suggesting that SpO2 may be a sensitive screening marker. That said, seven patients in our cohort had symptoms with entirely normal SpO2, suggesting that pulse oximetry alone is not enough and needs to be combined with direct symptom enquiry at each visit.

The four ABG-confirmed cases illustrate another important point: MetHb levels do not map neatly onto clinical severity [10,12]. The patient with the highest MetHb (7%) was symptomatic with low SpO2 and required dapsone discontinuation. The patient with a MetHb of 6.2% was completely asymptomatic and was identified only because SpO2 was routinely checked. Decisions about whether to continue or stop dapsone should therefore be guided by the overall clinical picture, including symptoms, SpO2, and functional impact, rather than by MetHb levels alone.

None of the risk factors evaluated in this study, such as G6PD status, dose, oxidizing drugs, antioxidants, or hemoglobin fall, were statistically significant, which likely reflects the small sample size rather than the absence of true associations. Esbenshade et al. (2011) [13] and Mitsides et al. (2014) [12] both found dose-related effects. One patient in our cohort was asymptomatic at 50 mg/day but developed clinical findings at 100 mg/day. While consistent with previous reports, this is a single anecdotal observation from which no dose-response relationship can be inferred.

Nine of the 12 clinically consistent patients had normal G6PD levels, confirming the mechanistic independence of methemoglobinemia from hemolysis: G6PD protects against the latter via the hexose monophosphate shunt, but methemoglobinemia is affected by a separate enzyme, CYB5R [1,2,13]. This distinction is well established in the literature but not always applied in practice. Clinicians who rely on a normal G6PD level to rule out dapsone-related hematologic toxicity may miss methemoglobinemia entirely.

The main limitations of this study are the retrospective design, the small cohort, and the absence of systematic ABG measurement. Retrospective symptom data are subject to documentation bias, and the clinical case definition, though pragmatic, is not a biochemical standard, so some cases may have been misclassified in either direction. Seven patients could not be classified owing to insufficient data; of these, five were asymptomatic with no documented SpO₂, and so represent likely non-cases. However, excluding them would raise the estimate to 51% (12/21), and we therefore retained all 28 patients to keep it conservative. The 13 patients lost to follow-up could not be assessed; as symptomatic patients may be more likely to default, their exclusion may have removed cases more often than non-cases, again suggesting our estimate is conservative. Most importantly, ABG co-oximetry, the diagnostic reference standard, was available in only four patients and was performed at the clinician's discretion rather than systematically. Consequently, the true biochemical prevalence could not be established, and the reported 43% reflects a clinical, not biochemical, definition. Because ABG was obtained only in clinically suspected patients and never in screen-negative ones, the sensitivity and specificity of the case definition cannot be quantified, and the 100% concordance in the four tested patients should not be read as diagnostic accuracy.

A further consequence is that asymptomatic methemoglobinemia may have gone undetected in some patients. One ABG-confirmed patient (MetHb 6.2%) was entirely asymptomatic, so some of the nine clinically negative patients may similarly have had undetected elevations, and the true biochemical prevalence may exceed the 43% identified clinically. Such subclinical elevations, however, may warrant monitoring rather than intervention, whereas the clinical case definition captures the functional burden that actually affects patients and that a purely biochemical threshold may overstate. These limitations would be addressed by a prospective study with protocolised ABG measurement. Nonetheless, the symptom- and SpO₂-based definition is feasible in outpatient dermatology and directly relevant to the outcomes that matter most to patients and clinicians. Quality of life was not formally assessed; references to functional impact are based on documented symptoms rather than a validated instrument.

## Conclusions

Clinical findings consistent with dapsone-induced methemoglobinemia may cause a significant burden in the dermatology outpatient setting. The predominant picture is one of mild-to-moderate disease: fatigue and exertional dyspnea on activity, with patients who appear normal at rest and have no classical signs such as cyanosis, tachycardia, or tachypnea. This presentation is easy to miss, easy to misattribute, and carries real implications for symptom burden and treatment adherence. Normal G6PD levels do not protect against methemoglobinemia, and G6PD testing cannot substitute for clinical surveillance. In all four patients where ABG MetHb was measured, the elevated biochemical result was consistent with the clinical assessment. This suggests, but does not confirm, the feasibility of a symptom- and oximetry-based approach to screening.

These findings provide a hypothesis-generating basis for a prospective study with systematic ABG MetHb measurement in dermatology outpatients starting dapsone. Such a study should aim to confirm the true incidence of biochemically defined methemoglobinemia in this population, identify MetHb thresholds that are clinically meaningful in this context, and clarify which patient and treatment factors predict who is at greatest risk. Until such data are available, a pragmatic approach would be to screen all patients on dapsone with symptom enquiry and pulse oximetry, as this is cost-effective and non-invasive. ABG could be reserved for those who screen positive, as it is invasive and not available at all clinics. However, the diagnostic performance of this strategy was not formally assessed in our study. Awareness of dapsone-induced methemoglobinemia among dermatologists is the first and most practical step in reducing its burden.

## Appendices

Other drugs associated with methemoglobinemia

Aminosalicylic acid (also called p-aminosalicylic acid or 4-aminosalicylic acid), clofazimine, antimalarials (chloroquine, primaquine), local anesthetics, menadione, metoclopramide, methylene blue, nitroglycerin and nitrates, phenacetin, phenazopyridine, rasburicase, quinones, and sulfonamides.
